# Dual anticancer activity of *Aspergillus nidulans* pigment and Ionizing γ-Radiation on human larynx carcinoma cell line

**DOI:** 10.1186/s12906-023-04162-x

**Published:** 2023-09-18

**Authors:** Hanaa Y. Ahmed, Eman M. El Gazzar, Nesreen Safwat, Monda M. M. Badawy

**Affiliations:** 1 The Regional Center for Mycology and Biotechnology (RCMB), Al-Azhar University, Cairo, Egypt; 2 Health Radiation Research Department, National Center for Radiation Research and Technology (NCRRT), Egyptian Atomic Energy Authority, Cairo, Egypt

**Keywords:** *Aspergillus nidulans*, Ionizing radiation, Anticancer activity, Apoptosis, P53, Caspase 3 and Bcl-2

## Abstract

**Background:**

Fungi are a readily available source of naturally generated colored compounds. These compounds might be used as radiosensitizers for treating cancer cells.

**Methods:**

*Aspergillus nidulans* was examined for its color-producing ability in Potato dextrose agar (PDA) broth medium. The pigment was characterized by Ultraviolet (UV) spectrophotometer and Gas Chromatography Mass Spectrometry (GC/MS). Pigment extracts from *A. nidulans* were studied for their cytotoxic effects on the growth of human larynx carcinoma cell line (HEp-2) with or without exposure to γ-radiation at three different doses (5, 10, and 15 Gy). *A. nidulans* pigment cytotoxic activity was tested against normal Vero cells. Cell apoptosis was studied using flow cytometry. Gene expression of P53, Caspase 3 and Bcl-2 were quantified.

**Results:**

Ultraviolet spectrum and GC/MS revealed the ability of *Aspergillus nidulans* to produce Rhodopin pigment. HEp-2 cells treated with *A. nidulans* pigment only give IC_50_ about 208 µg/ml. In contrast, when treated with the pigment +10 Gy γ-radiation, it give about 115 µg/ml. However, for normal cells, lower cytotoxic activity was detected. Treatment with pigment (208 g/mL) caused about 50% ± 1.0 total apoptosis level and gene expression of P53: 2.3 fold and Caspase 3: 1.84 fold in respect to untreated HEp-2), while Bcl-2 was decreased (Bcl-2: 0.63 fold in respect to untreated HEp-2). Furthermore, treated with pigment (115 µg/mL) + 10Gy caused about 47.41% ± 1.7 total apoptosis level and P53: 2.53 fold and Caspase 3: 2.0 fold in respect to untreated HEp-2, while Bcl-2 was downregulated (Bcl-2: 0.61 fold in respect to untreated HEp-2).

**Conclusion:**

This study concluded that the anti-cancer activity of *Aspergillus nidulans* pigment was enhanced by ionizing radiation at 10 Gy, as well as its low cytotoxic activity against normal Vero cells.

## Introduction

There are 211,000 new instances of laryngeal cancer (LC) each year, with an expected 126,000 deaths [1]. LC is a type of cancer that often manifests in adults in their sixth or seventh decade with a history of substantial tobacco and alcohol usage [2]. Radiotherapy is one of the most effective cancer treatment methods. Approximately 60% of all cancer patients are treated with ionizing radiation (IR) [3].

Radiotherapy targets particular areas of the body with exact quantities of radiation. In radiotherapy (RT), ionizing radiation such as X-rays, gamma-rays, electrons, neutrons, and charged particles are employed. This radiation may destroy cells by directly interacting with critical targets and indirectly producing free radicals [4, 5].

The majority of radiosensitizers are used to radiosensitize cancer cells. Radiosensitizers are substances that, when used together with radiation, sensitize cancer cells, resulting in greater tumor inactivation by increasing the molecular absorption of free radicals produced by radiation damage [6].

Fungi are among the many microorganisms that produce pigments that may be utilized safely as natural antioxidants [7]. Fungal pigments are attracting the interest of pharmaceutical companies as potential drug sources for treating a wide range of fatal illnesses, including cardiovascular disorders, Alzheimer's disease, human carcinomas (hepatoma, breast, lung, colorectal, gastric, pancreatic, leukemia, hematopoietic, renal cell, and other cancers), infectious diseases, and parasitic diseases such as malaria. Among the fungal pigments, are carotenoids. All Carotenoids have excellent antioxidant properties and aid in the preventing of cancer, diabetes, immune system weakness, cardiovascular disease, and cataract development [8]. Numerous natural carotenoids, in addition to β-carotene, have been demonstrated to have anticarcinogenic potential, with some exhibiting more potent activity than β-carotene [9].

Apoptosis occurs automatically in malignant tumors, significantly slowing their growth, and it takes place in cancers that react to irradiation, cytotoxic chemotherapy, heating, and hormone ablation [10]. P53 works as a genome protector and is a key regulator of cell proliferation, growth, and transformation. The P53 tumor suppressor gene is altered in more than half of all human malignancies, and its oncogenic activity is due to its ability to interfere with P53-dependent apoptosis via a dominant negative mechanism [11].

Inducing apoptosis in cancer cells by targeting essential apoptosis regulators remains a promising and successful technique for drug discovery and the developing of novel anticancer medicines. Caspase 3 is an enzyme that is required for apoptosis to occur. The caspase (cysteine-aspartate protease) family is one of six protease families whose activities are closely connected to the processes of programmed cell death (apoptosis, pyroptosis, and necroptosis) and inflammation [12]. Caspase 3 activity indicates irreversible cell death [13]. Bcl-2 (B cell lymphoma 2) is an anti-apoptotic gene that plays a crucial function in apoptosis regulation. A decrease in Bcl-2 expression results in apoptosis [14].

In this study, we enhanced the anticancer activity of natural pigment derived from *A. nidulans* by combining it with gamma rays at different doses (5, 10, and 15 Gy) to test its effectiveness against HEp-2 cells.

## Materials and methods

### Materials

- Fungal strains: The test organism *Aspergillus nidulans* 002018(2) was collected from the Regional Center for Mycology and Biotechnology (RCMB) et al.-Azhar University.
- FITC Annexin V Apoptosis Detection Kit I (BD Biosciences, cat. No. 556547).
- RNeasy Mini Kit (Qiagen, Cat. No. 74104).
- Revert Aid First Strand cDNA synthesis kit (Thermo Scientific, Cat. No. K1622).
- QIAGEN QuantiTect® SYBR® Green PCR kit (Qiagen, Cat. No. 204143).

### Qualitative screening for pigment production

The color-producing capacity of the test organism *Aspergillus nidulans* was investigated. Sterilized Potato Dextrose agar medium (pH 7.0) was cooled to 45 °C and aseptically transferred to a pre-sterile Petri plate. After adequate mixing and solidification, the fungal species was injected at the center of the pour-plated plates with the medium in triplicates The plates were incubated in the incubator for 7 days at 28 ± 1 °C. Fungal colonies' growth patterns and pigment-producing abilities were tested on different days (after 1, 3, 5, and 7). After each incubation period, the colonies were examined for hyphal growth and pigment generation [15].

### Production of microbial pigments

The organism under study, *Aspergillus nidulans*, was placed in a medium containing PDA broth (pH 7.0). For 15 days, the infected flasks were housed in an incubator at 28 ± 1 °C under stable conditions and darkness, with occasional inspection at varied intervals.

### In vitro pigment extraction and assay

According to the technique presented by Velmurugan et al. (2010) [16], The pigment was obtained from biomass. Five grams of the cooled fresh mycelial mat were gently removed and rinsed with sterile distilled water. The water was changed several times during each wash until the water flow became clear. Then ethanol (90%) was added to the test tube, the ratio of ethanol to water was 1:10 (10 mL ethanol per gram of biomass). They were immersed in ethanol and then in a boiling water bath to isolate the colors from the mycelial mats. The mycelial mat was homogenized to produce a suspension with a little acid-washed and oven-sterilized sand. This was done to eliminate any remaining colors in the biomass or mycelial mats. The slurry was filtered using Whatman No. 1 filter paper after sitting for 15 min and running through an orbital shaker set to 200 rotations per minute for one hour. The absorbance of the colored extract or pigment produced was evaluated at 500 nm using UV–visible Spectroscopy Analysis [17].

### GC–MS analysis and conditions

A Trace GC1310-ISQ mass spectrometer (Thermo Scientific, Austin, TX, USA) with a direct capillary column TG-5MS (30 m × 0.25 mm × 0.25 m film thickness) was used to analyze the chemical composition of materials. The column oven temperature was initially kept at 50 °C before increasing by 5 °C/min to 230 °C for 2 min, increased at 30 °C/min to a final temperature of 290 °C and held for 2 min. The injector and MS transfer line temperatures were fixed at 250 °C and 260 °C, respectively; helium was utilized as a carrier gas at a constant flow rate of 1 mL/min. The solvent delay was 3 min, and 1 µl diluted samples were injected automatically using an Autosampler AS1300 linked with a GC in split mode. In full scan mode, mass spectra were obtained at 70 eV ionization voltages over m/z 40–1000. The temperature of the ion source was fixed at 200 °C. The components were identified by comparing their retention durations and mass spectra to the mass spectral databases WILEY 09 and NIST 11.

### Gamma irradiation

Cell lines were exposed to different doses of 5, 10, and 15 Gy by using cesium 137 as a source of gamma radiation (Gamma cell − 40 Canadian, Activity 3032 Ci, Dose rate: 0.675 rad/second at the time of experiment at room temperature), At NCRRT, Cairo, Egypt.

### Anticancer activity using MTT assay

Cytotoxic effects on HEp-2 (Human Larynx carcinoma cell line) were studied in three groups:

- Group (1): HEp-2 cells were incubated for 24 h in a tissue culture medium after exposure to different γ-radiation doses (5, 10, and 15Gy).
- Group (2): HEp-2 cells were incubated for 24 h in a tissue culture medium with different dilutions of *A. nidulans* pigment ranging from 15.63 μg/mL to 500 μg/mL.
- Group (3): HEp-2 cells were incubated for 24 h in a tissue culture medium with different dilutions of *A. nidulans* pigment ranging from 15.63 μg/mL to 500 μg/mL after exposure to different γ-radiation doses (5, 10, and 15Gy).

The plates were incubated for 24 h at 37°C in a humidified incubator with 5% CO_2_. Then, the culture supernatant was replaced with fresh media. The cells in each well were then treated for 4 h at 37°C with 100 µL of MTT solution (5 mg/mL). The MTT solution was then withdrawn, and 100 µL of DMSO was added to each well. A microplate ELISA reader (SunRise TECAN, USA) was used to measure absorbance at 570 nm [18].

### Cytotoxicity on normal cell

The MTT assay assessed the viability of normal Vero cells after treatment with pigment with or without irradiation at 10 Gy. Vero (Normal monkey kidney cell line) was cultivated in RPMI media for 24 h until confluence, then treated with various substances and incubated for another 24 h at 37°C. The MTT assay was applied as in the previous experiment.

### Flow cytometry analysis of cell apoptosis

After 24 h of incubation with the extracted pigment with or without exposure to 10 Gy ionizing γ-radiation, apoptosis detection was investigated using FITC Annexin V Apoptosis Detection Kit I (BD Biosciences, cat. No. 556547). Cells were collected and washed twice with cold PBS before being resuspending in 1X Binding Buffer at a concentration of 1 × 10^6^ cells/ml. 100 µl of the solution (1 × 10^5^ cells) was transferred to a clean tube, then mixed with 5 µl of FITC Annexin V and 5 µl of PI. Cells were gently mixed and incubated in the dark for 15 min at room temperature (25 °C). Each tube was then filled with 400 µl of 1X Binding Buffer. After 30 min, the cells were analyzed with a flow cytometer (BD Accuri™ C6 Plus).

### Determination of gene expression of P53, Caspase 3 and Bcl-2

Total RNA was extracted from cell suspension using the RNeasy Mini Kit (Qiagen, Cat. No. 74104) following the manufacturer's instructions. RNA concentration and purity were determined using Nabi- UV/Vis Nano Spectrophotometer, MicroDigital co.,Ltd.,Korea. The RNA was used as a template for first-strand complementary deoxyribonucleic acid (cDNA) production using the Revert Aid First Strand cDNA synthesis kit (Thermo Scientific, Cat.No. K1622). The Rotor-Gene Q RT-PCR cycler from QIAGEN was used for quantitative RT-PCR. The cDNA (3 μl) was amplified in a 25 μl reaction mixture using the QIAGEN QuantiTect® SYBR® Green PCR kit (Cat. No.204143) and primers specified in Table 1. The sequences of P53, Caspase 3, and Bcl-2 oligonucleotide primers were chosen in accordance with Mansour et al. (2021) [19]. After a 15-min Taq activation step at 95 °C (hot start), reactions were subjected to 45 cycles of 15 s of denaturation at 94 °C, 30 s of annealing at 60 °C, and 30 s of extension at 72 °C. ΔΔCt method was used to determine the relative expression of the real-time reverse transcriptase PCR products. This approach estimates a relative expression to the housekeeping gene using the equation fold induction = 2^−(ΔΔCt)^. Where ΔΔ Ct = Ct [gene of interest (unknown sample)-Ct housekeeping gene (unknown sample)]—[Ct gene of interest (calibrator sample)—Ct housekeeping gene (calibrator sample)] [20].

**Table 1:** Primer sequences for the genes amplified

Gene	Strand	Sequence 5`—3`	Product length (bp)	Ref. Seq
**P53**	**F**	**CGCTTCGAGATGTTCCGAGAG**	**102**	**NM_000546**
	**R**	**CTTCAGGTGGCTGGAGTGAG**		
**Caspase 3**	**F**	**GAAGCGAATCAATGGACTCTGG**	**127**	**NM_004346**
	**R**	**GACCGAGATGTCATTCCAGTGC**		
**Bcl-2**	**F**	**TTGATGGGATCGTTGCCTTATGC**	**107**	**NM_000657**
	**R**	**CAGTCTACTTCCTCTGTGATGTTG**		
**GAPDH**	**F**	**GACCTGACCTGCCGTCTAG**	**98**	**NM_002046**
	**R**	**TAGCCCAGGATGCCCTTGAG**		

### Statistical analysis

The results were displayed as mean ± standard deviation of the mean (S.D.). One-Way ANOVA was used to analyze the data, following the Tukey–Kramer multiple comparison test. The statistical analysis and graphical presentations have been carried out using Graph Prism software, version 5, Inc., USA. For all statistical tests, the level of significance was set at *P* < *0.05*.

## Results

### Qualitative screening for pigment production

**Fig. 1:**
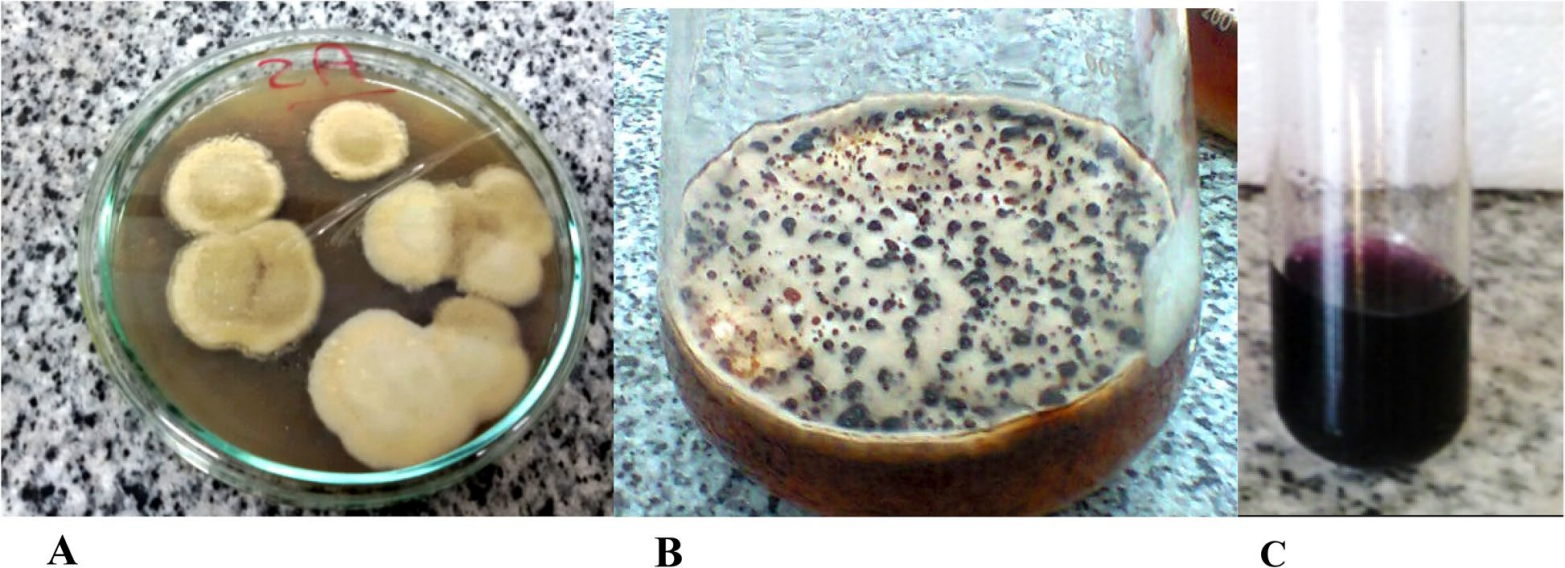
Production of Aspergillus nidulans pigments on; (**A**) PDA media and (**B**) PDA broth media. **C** Ethanol extract

On PDA plates, *Aspergillus nidulans* capacity for pigment production was analyzed to determine its efficacy (Fig. 1A). The plate method, on the other hand, was not appropriate for quantifying Pigment. As a result, the strains were further investigated by screening them on the PDA broth medium, where the highest pigment production was found (Fig. 1B). Pigment with dark red was extracted using 90% ethanol (Fig. 1C).

### UV–visible spectroscopy analysis

**Fig. 2:**
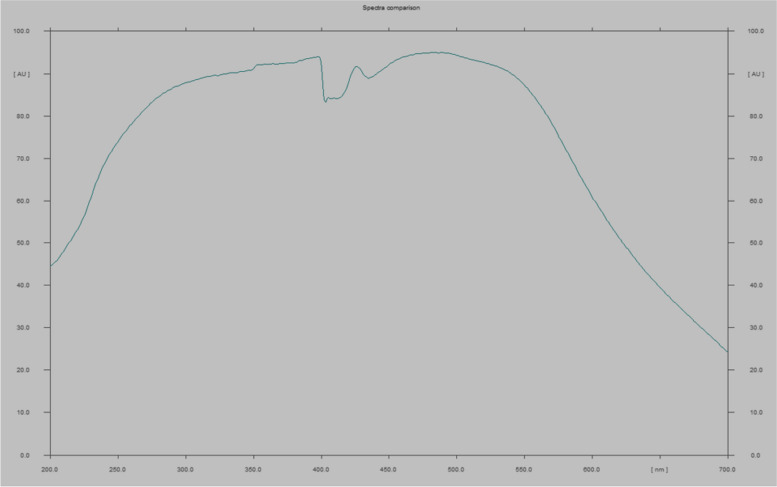
UV–visible absorption spectrum of pigment produced from Aspergillus nidulans

The ultraviolet spectrum revealed the presence of beneficial color pigments produced by the *Aspergillus nidulans* at peaks from 450 to 550nm (Fig. 2).

### GC–MS analysis

**Fig. 3:**
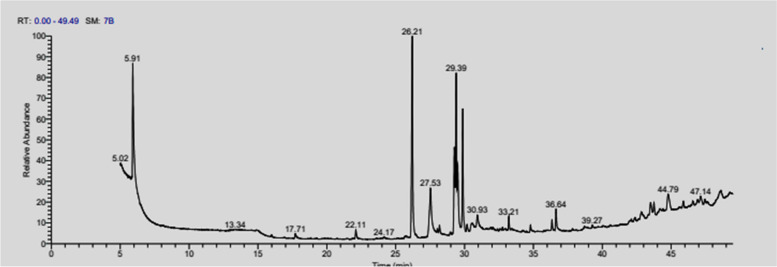
GC–MS chromatogram of *Aspergillus nidulans* extract

The ethanol extract of *Aspergillus nidulans* was separated by GC/MS to detect the extract's main constituents. Table 2 and Fig. 3 represented the compounds in the extract are Dimethylsulfoxonium formylmethylide, Dodecanoic acid, Methyl ester, Methyl tetradecanoate, Hexadecanoic acid, Methyl ester, 5-(7a-Isopropenyl-4,5-dimethyl-oct ahydroinden-4-yl)-3-methyl-penta-2,4-dien-1-ol, 9-Octadecenoic acid, methyl ester, cis-5,8,11,14,17-Eicosapentaenoic acid, Docosanoic acid, Methyl ester, Diisooctyl phthalate, Ethyl iso-allocholate, 1H-2,8a-Methanocyclopenta[a]cycl opropa[e]cyclodecen-11-one, 1a,2,5,5a,6,9,10,10a-octahydro-5,5a,6-trihydroxy-1,4-bis(hydroxymethy l)-1,7,9-trimethyl-, [1S-(1à,1aà,2à,5á,5aá,6á,8aà,9à,10 aà)]-, Stigmast-5-en-3-ol, (3á,24S)-, Rhodopin. So, the GC/MS revealed The ability of *Aspergillus nidulans* to produce Rhodopin pigment.

**Table 2:** Chemical constitutes of *Aspergillus nidulans* extract separated by GC–MS

RT	Chemical name	Formula	Peak area (%)
5.91	Dimethylsulfoxonium formylmethylide	C_4_H_8_O_2_S	10.66
17.71	Dodecanoic acid, methyl ester	C_13_H_26_O_2_	0.50
22.11	Methyl tetradecanoate	C_15_H_30_O_2_	0.91
26.21	Hexadecanoic acid, methyl ester	C_17_H_34_O_2_	21.50
27.53	Hexadecanoic acid	C_16_H_32_O_2_	5.12
28.18	5-(7a-Isopropenyl-4,5-dimethyl-oct ahydroinden-4-yl)-3-methyl-penta-2,4-dien-1-ol	C_20_H_32_O	0.73
29.26	9,12-Octadecadienoic acid (Z,Z)-, methyl ester	C_19_H_34_O_2_	8.25
29.39	9-Octadecenoic acid, methyl ester, (E)	C_19_H_36_O_2_	17.39
29.50	9-Octadecenoic acid, methyl ester, (E)-	C_19_H_36_O_2_	6.15
29.87	Octadecanoic acid, methyl ester	C_19_H_38_O_2_	10.53
30.18	cis-5,8,11,14,17-Eicosapentaenoic acid	C_20_H_30_O_2_	0.76
30.51	9-Octadecenoic acid (Z)-	C_18_H_34_O_2_	1.34
30.93	9-Octadecenoic acid (Z)	C_18_H_34_O_2_	1.40
33.21	Eicosanoic acid, methyl ester	C_21_H_42_O_2_	1.15
34.79	Ethanol, 2-(9-octadecenyloxy)-, (Z)-	C_20_H_40_O_2_	0.64
36.35	Docosanoic acid, methyl ester	C_23_H_46_O_2_	0.94
36.64	Diisooctyl phthalate	C_24_H_38_O_4_	1.95
42.85	Ethyl iso-allocholate	C_26_H_44_O_5_	0.87
43.51	1H-2,8a-Methanocyclopenta[a]cycl opropa[e]cyclodecen-11-one, 1a,2,5,5a,6,9,10,10a-octahydro-5,5a,6-trihydroxy-1,4-bis(hydroxymethy l)-1,7,9-trimethyl-, [1S-(1à,1aà,2à,5á,5aá,6á,8aà,9à,10 aà)]-	C_20_H_28_O_6_	1.52
43.75	Stigmast-5-en-3-ol, (3á,24S)-	C_29_H_50_O	1.07
44.78	Ethyl iso-allocholate	C_26_H_44_O_5_	2.71
45.89	Ethyl iso-allocholate	C_26_H_44_O_5_	0.79
47.15	Rhodopin	C_40_H_58_O	1.11
47.44	Rhodopin	C_40_H_58_O	0.54
48.61	Rhodopin	C_40_H_58_O	1.50

Rhodopin (1,2-dihydro-ψ,ψ-carotene-1-ol) is a carotenoid. Carotenoids, or tetraterpenoids, are organic pigments that can be yellow, orange, or red. These pigments are produced by a variety of microorganisms, including fungi.

### Antitumor activity

**Fig. 4:**
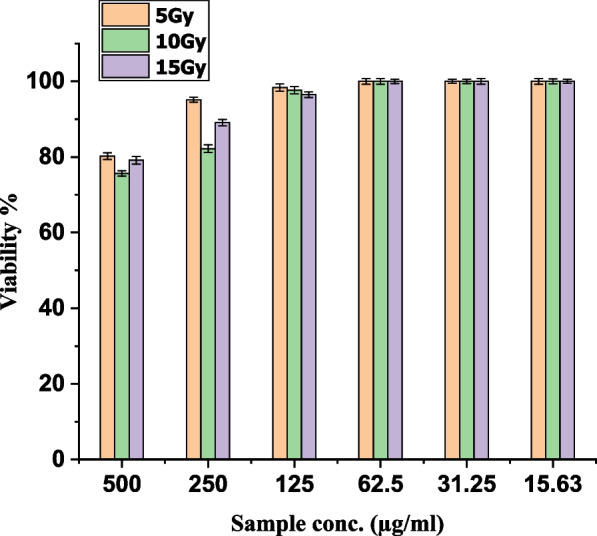
Effect of different γ-radiation doses on HEp-2 cells

**Fig. 5:**
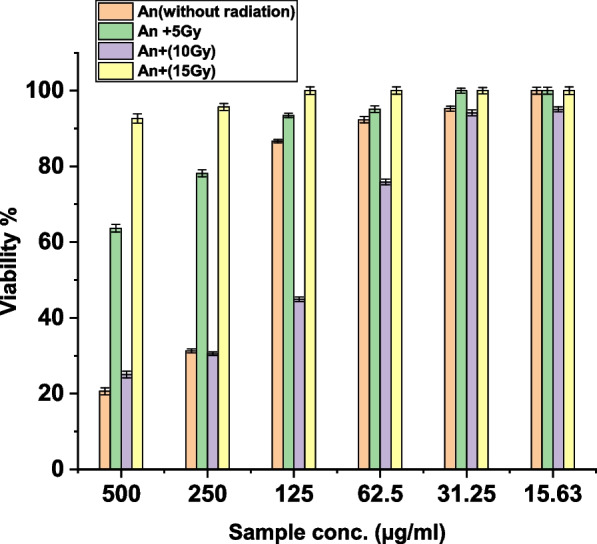
The antitumor activity of the pigment extracts of *A. nidulans* (An) without irradiation and with 5, 10, and 15 Gy γ-irradiation after 24 h of treatment against HEp-2 cells

Using MTT assay, the in vitro cytotoxic effect on the growth of HEp-2 (Human Larynx carcinoma cell line) after exposure to three doses of γ- radiation (5, 10, and 15 Gy) was studied in comparison with the cytotoxic effect on the growth of HEp-2 with *A. nidulans* pigment ranging from 500 μg/mL to 15.63 μg/mL. In addition to the cytotoxic effect of both gamma rays and *A. nidulans* pigment. The highest antitumor activity was recorded with *A. nidulans* pigment plus 10 Gy gamma radiation compared to *A. nidulans* pigment alone. At the same time, no antitumor activity was observed from the *A. nidulans* pigment with 5 and 15 Gy γ-radiation and the *irradiated* control cells at 5,10, and 15 Gy without Pigment (Figs. 4,5). The best IC_50_ values were recorded at a dose of 10 Gy, about 115 μg/mL. In contrast, IC_50_ values of un-irradiated HEp-2 cells with *A. nidulans* pigment were measured at 208 μg/mL (Table 3).

**Table 3:** IC_50_ values of *A. nidulans* pigment extracts (An) and/or irradiation on HEp-2 cells

IC_50_ (µg/mL)	
5Gy	> 500
10 Gy	> 500
15 Gy	> 500
An (without irradiation)	208 ± 0.46
An + 5 Gy	> 500
An + 10 Gy	115 ± 0.76
An + 15 Gy	> 500

### Cytotoxic activity on normal vero cells

From the results in Table 4, it was observed that the cytotoxic activity of the pigment without irradiation on normal Vero cells was lower than on HEp-2 cells in which IC_50_ value was increased from 208 µg/mL to 223 µg/mL. Also, the IC_50_ value of the pigment with irradiation at 10 Gy on Vero cells was increased from 115 µg/mL to 215 µg/mL. No cytotoxic activity was observed on normal Vero cells after exposure to 10 Gy without pigment.

**Table 4:** IC_50_ values of the pigment extracts of *A. nidulans* (An) with or without irradiation on normal Vero cells

IC_50_ (µg/mL)	
10Gy	> 500
An (without irradiation)	223 ± 0.53
An + 10 Gy	215 ± 0.32

### Apoptosis by flow cytometry

**Fig. 6:**
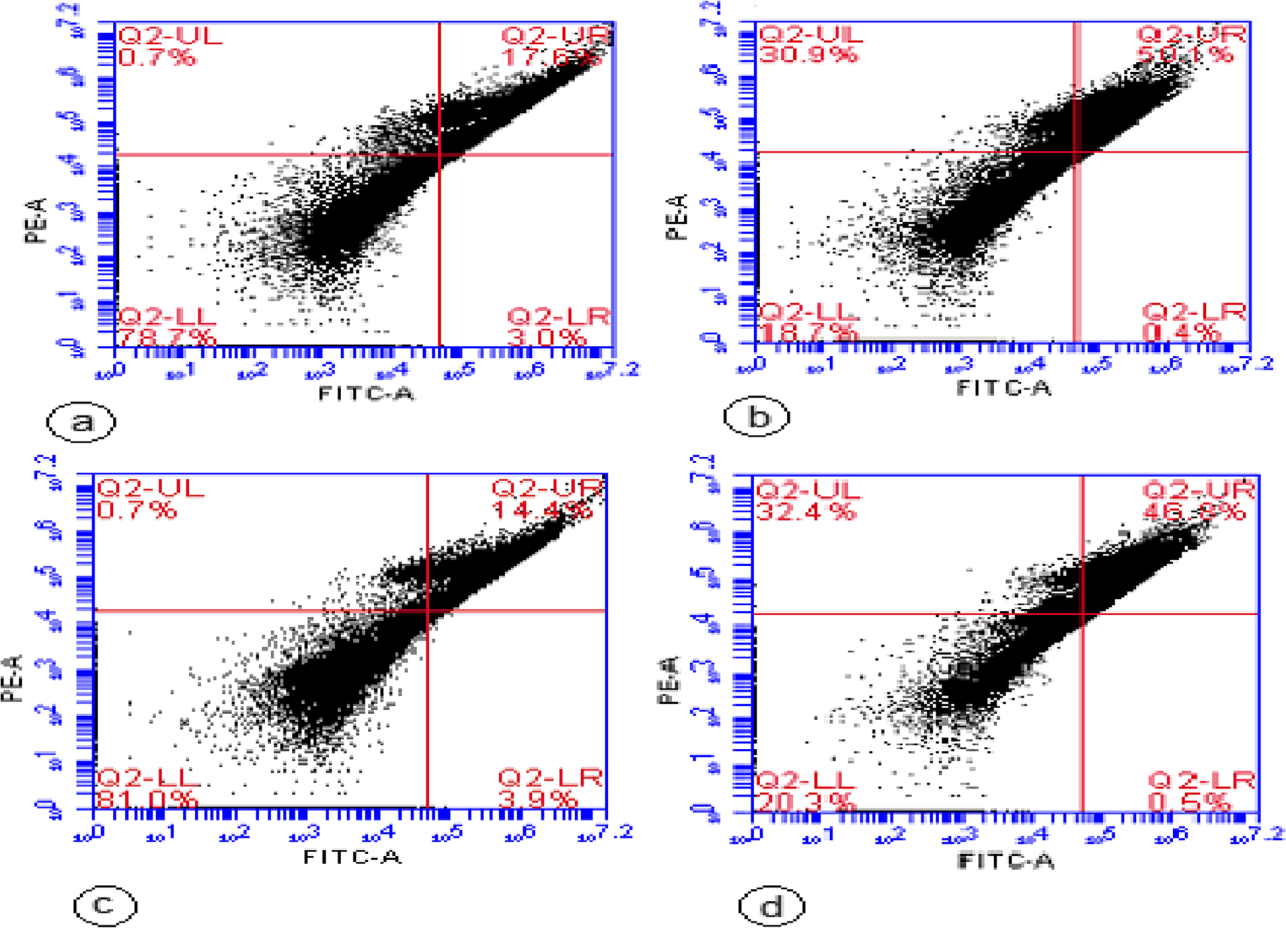
Showing flow cytometer results. Where (**a**) control (HEp-2 without treatment), (**b**) treated with the pigment extracted from *A. nidulans* (208 µg/mL), (**c**) exposed to 10 Gy ionizing γ-radiation, and (**d**) treated with the pigment extracted from *A. nidulans* (115 µg/mL) + 10 Gy ionizing γ-radiation

**Fig. 7:**
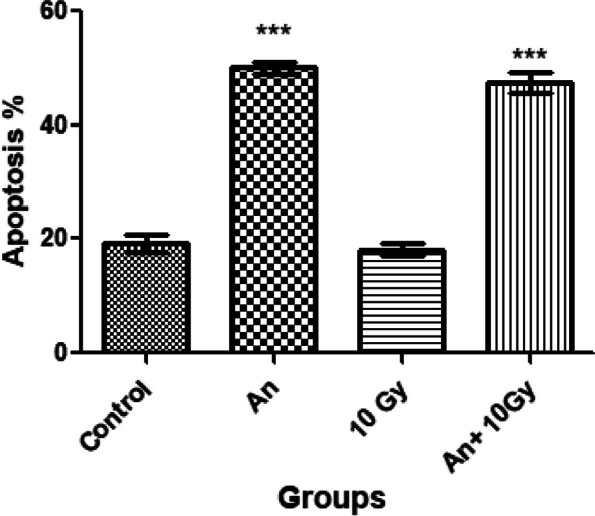
Apoptosis level (early and late apoptotic cells) in HEp-2 cells treated with 208 g/mL *A. nidulans* pigment (An), 10 Gy ionizing radiation, and 115 µg/mL *A. nidulans* pigment + 10 Gy of ionizing radiation (An + 10 Gy) using fow cytometry. The results are shown as mean ± SD in triplicate (*n* = 3). Where, *** Extremely significant compared to untreated cells at *p* ≤ 0.05

The results obtained from Annexin V/Propidium Iodide flow cytometry assay are shown in Fig. 6. As it turns out in Fig. 7, the total apoptosis level (early and late apoptotic cells) were signifcantly increased in HEp-2 cells treated with 208 g/mL *A. nidulans* pigment (50% ± 1.0) and HEp-2 cells treated with 115 µg/mL *A. nidulans* pigment + 10 Gy of ionizing radiation (47.41% ± 1.7) compared to the untreated cells (*p* < *0.05*).

### Gene expression of P53,Caspase 3 and Bcl-2

**Fig. 8:**
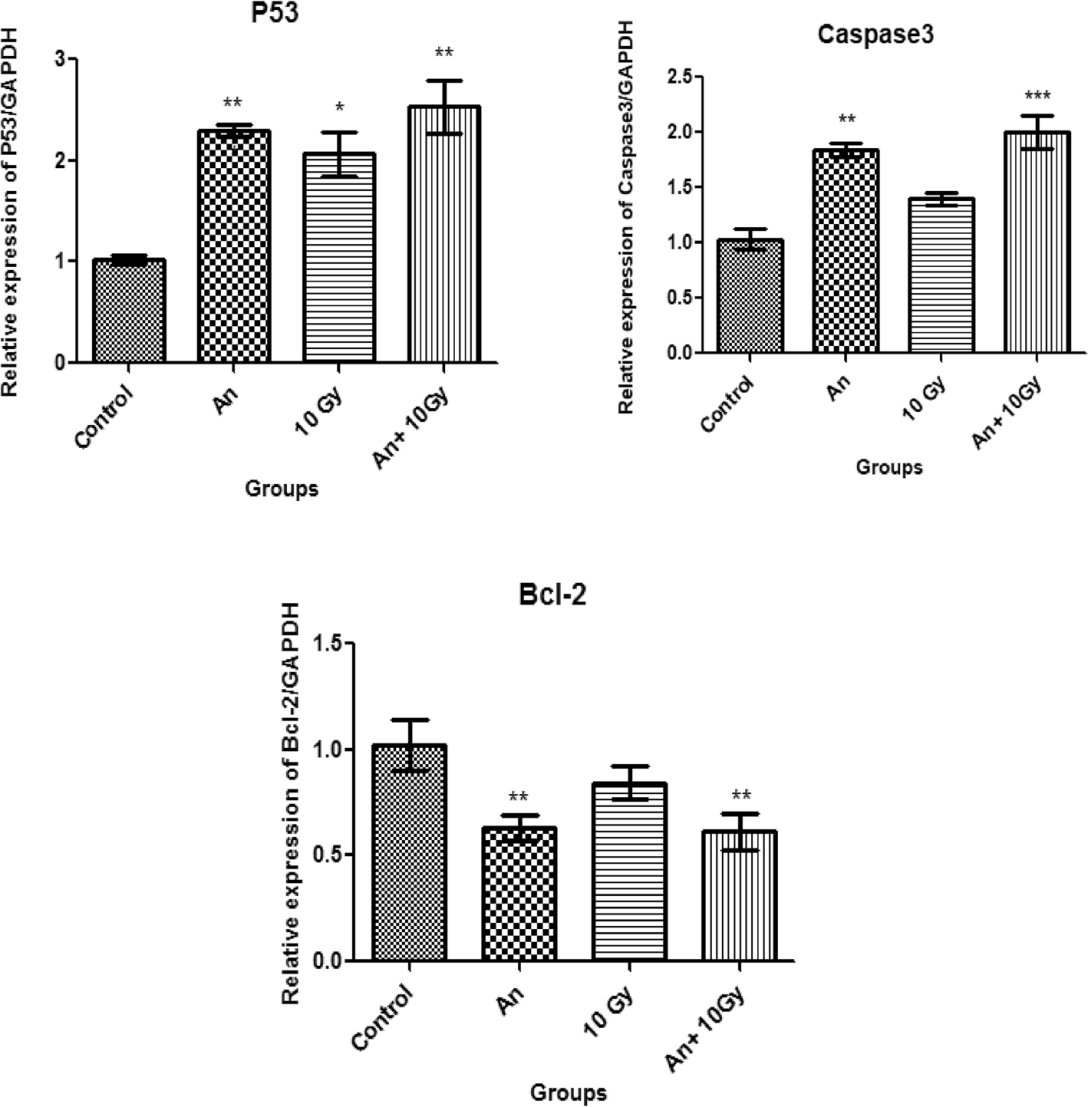
Gene expression of P53, Caspase 3 and Bcl-2

Treated with the pigment extracted from *A. nidulans* (208 µg/mL) manifested a significant (*P* ≤ 0.05) increase in P53 and Caspase 3 genes expression (P53: 2.3 fold and Caspase 3: 1.84 fold in respect to untreated HEp-2), while Bcl-2 was downregulated (Bcl-2: 0.63 fold in respect to untreated HEp-2). Moreover, treatment with the pigment extracted from *A. nidulans* (115 µg/ml) and exposure to 10 Gy ionizing γ-radiation results in higher expression than the pigment extracted from *A. nidulans* only with a higher dose (P53: 2.53 fold and Caspase 3: 2.0 fold in respect to untreated HEp-2), while Bcl-2 was downregulated (Bcl-2: 0.61 fold in respect to untreated HEp-2). (Table 5 and Fig. 8).

**Table 5:** Gene expression of P53, Caspase 3 and Bcl-2

	**P53**	**Caspase 3**	**Bcl-2**
Control (untreated HEp-2)	1.02 ± 0.08	1.03 ± 0.15	1.02 ± 0.12
An	2.3 ± 0.1^**^	1.84 ± 0.11^**^	0.63 ± 0.06^**^
10 Gy	2.1 ± 0.38^*^	1.4 ± 0.10	0.84 ± 0.08
An + 10 Gy	2.53 ± 0.45^**^	2.0 ± 0.26 ^***^	0.61 ± 0.09^**^

Each value represents the mean ± standard deviation

^*^Significant difference versus control group at *p* ≤ 0.05

^**^Very significant versus control group at *p* ≤ 0.05

^***^Extremely significant versus control group at *p* ≤ 0.05

## Discussion

Since the discovery of penicillin, fungi have been one of the most important sources of therapeutic compounds, including antibiotics, anticancer drugs, and cholesterol-lowering pharmaceuticals [21]. Many fungi are readily generated in lab conditions, in addition to their vast dispersion and ability to create secondary metabolites such as pigments with medicinal significance. Both fundamental research and the pharmaceutical business are interested in the potential of large-scale manufacturing [22]. Fungi create pigments with various structures and colors. These fungi include Monascus, *Aspergillus*, *Penicillium*, and *Paecilomyces* [23] pigments are classified into four types: carotenoids, melanins, polyketides, and azaphilones (polyketide derivatives) [24, 25]. Natural chemicals with anticancer potential are promising sources for treating various malignancies. One of these natural substances is fungi pigments [26].

In accordance with prior research, the current study discovered *Aspergillus nidulans'* ability to form colors on broth PDA medium. According to Saitou and Nei (1987) [27], there is a lot of promise in the amazing color spectrum of pigments generated by ascomycetous fungus in the red and yellow spectra. *Aspergillus* and *Penicillium* genera microbes have also been studied as possible sources of natural pigments [28].

In the current study, the crude extract of the culture of *Aspergillus nidulans* was analyzed on GC/MS. Different compounds were separated from GC; among these compounds, Rodopin. Rhodopin is a carotenol with the chemical formula 1,2-dihydro-psi,psi-carotene with a hydroxyl group at the C-1 position. It acts as a metabolite in the bodies of bacteria. This substance also contains carotenol and tertiary alcohol. Rhodopin is a naturally occurring chemical generated by *Rhodomicrobium vannielii*, *Afifella marina*, and perhaps more species for which data is currently available. Carotenoids are tetraterpenoids that are organic pigments that are yellow, orange, and red in color. Plants, algae, bacteria, and fungi produce these colors [29].

Campanhol et al. (2023) reported that the identified spore pigment ascoquinone and asperthecin of *A. nidulans* protect ascospores from UV light which can absorb light in the UV range [30]. as well Han et al. (2020) and Zhang et al. (2020) Reported that *Aspergillus nidulans* bear pigmented ascospores which protect the fungi from UV radiation. The red pigment was a polyketide-derived dimeric hydroxylated anthraquinone [31, 32].

Furthermore, radiation treatment eliminates tumor cells while causing little injury to healthy organs. Depending on the kind of radiation, dosage, fractionation rate, and target organ, ionizing radiation can biologically kill cancer cells [33]. Radiation treatment targeting cancer cells can be enhanced without hurting healthy cells by using radiosensitizers for tumor cells and radioprotectors for normal cells [6].

According to the present study, the pigment extracts of *A nidulans* were tested for anticancer activity against HEp-2 cell line. Compared to *A. nidulans* pigment alone, *A. nidulans* pigment +10 Gy γ-radiation demonstrated the best anticancer activity. Simultaneously, no anticancer activity was found from *A. nidulans* pigment with 5 and 15 Gy γ-radiation or irradiated cells with 5, 10, and 15 Gy without pigment. The best IC_50_ values, around 115 g/mL, were obtained at a dosage of 10 Gy with *A. nidulans* pigment. The IC_50_ value of *A. nidulans* pigment in unirradiated HEp-2 cells was 208 g/mL. Gonçalves et al. (2015) revealed the mutagenicity and cytotoxicity of melanin pigment extracted from *A. nidulans* after its exposure to liver S9 enzymes [34]. Also, siderophore pigment extracted from *A. nidulans* was observed to have activity against HepG-2 [35].

Various treatment techniques combining radiotherapy and chemotherapy have been advocated as an alternative to primary surgery, which often entails a total laryngectomy, in locally advanced laryngeal and hypopharyngeal carcinoma to preserve laryngeal function. However, a viable alternative must exhibit efficacy equivalent to surgical intervention followed by radio(chemo)therapy [36]. So, the increased inhibitory activity after using *A. nidulans* pigment combined with radiation at 10 Gy can be supported by the previous studies, which concluded that radiotherapy could be enhanced by using radiosensitizing agents [37].

The results of the present study also suggest the low cytotoxic effect of *A. nidulans* pigment on normal Vero cells with IC_50_ value of 223 μg/mL. A decrease in the effect on the normal cells than cancer cells was observed after exposing the cells to the pigment and irradiation at a dose of 10 Gy with IC_50_ value 215 μg/mL. Ionizing radiation is used for killing cancer cells. However, it is also toxic to normal cells and causes cellular damage and different side effects. So, naturally occurring compounds have been shown to be non-toxic or low toxic on normal cells and are inexpensive and effective. Natural compound can be used as radioprotectors to inhibit radiation-induced toxicities or decreased toxicity on normal cells [38].

The present study showed that HEp-2 cell line treatment with *A. nidulans* pigment at a 208 µg/mL dose significantly increased apoptosis. Moreover, HEp-2 cell line treated with pigment at a dose of 115 µg/mL and exposed to 10 Gy of γ-radiation results in nearly similar apoptosis percentages. Gene expression of P53 and Caspase 3 after treatment with 208 µg/mL *A. nidulans* pigment manifested a significant increase, while Bcl-2 gene expression decreased (P53: 2.3 fold, Caspase 3: 1.84 fold, and Bcl-2: 0.63 fold in respect to untreated HEp-2). Moreover, treatment with 115 µg/mL *A. nidulans* pigment + 10 Gy ionizing γ-radiation results in higher expression for P53 and Caspase 3 and lower expression for Bcl-2 (P53: 2.53 fold, Caspase 3: 2.0 fold, and Bcl-2: 0.61 fold in respect to untreated HEP-2). Carotenoids are being studied extensively for their anticancer properties [39]. Carotenoids are a type of fungal pigment and are well-recognized in the normal cellular environment for their antioxidant and anticancer properties [40]. However, in cancer cells with a high intracellular ROS level, carotenoids may behave as powerful pro-oxidant agents and induce ROS-mediated apoptosis [41]. Carotenoids stimulate ROS production, which is followed by mRNA expression of caspase-3, -7, and -9, Bax, and p53, with concomitant inhibition of antiapoptotic Bcl-2. These events cause nuclear condensation, mitochondrial membrane potential loss, caspase-3 protein activation, and nuclei DNA breakage [42]. P53 regulates the cell cycle and is essential in ensuring that damaged cells are destroyed through apoptosis. Bcl-2 is an antiapoptotic protein that is a member of the B-cell lymphoma-2 (BCL-2) family. Bcl-2 inhibits apoptosis by attaching to proapoptotic members and inhibiting their activity [43].

Flowcytometery results showed that HEp-2 cell line treatment with 208 µg/mL *A. nidulans* pigment manifested a significant increase in apoptosis ((50% ± 1.0). Moreover, HEp-2 cell line treated with 115 µg/mL *A. nidulans* pigment and exposed to 10 Gy of γ-radiation results in nearly similar apoptosis percentage (47.41% ± 1.7).

The current study concluded that the anticancer activity of natural pigment derived from *A. nidulans* was enhanced by combining gamma rays at a dose of 10 Gy against HEp-2 cells with low cytotoxic activity against normal Vero cells.

## Data Availability

All data generated or analyzed during this study are included in this article.
